# NeuroConv: Streamlining Neurophysiology Data Conversion to the NWB Standard

**DOI:** 10.25080/cehj4257

**Published:** 2025-07-10

**Authors:** Heberto Mayorquin, Cody Baker, Paul Adkisson-Floro, Szonja Weigl, Alessandra Trappani, Luiz Tauffer, Oliver Rübel, Benjamin Dichter

**Affiliations:** 1 CatalystNeuro, Center for Open Neuroscience; 2 Dartmouth College, Center for Open Neuroscience; 3 Lawrence Berkeley National Laboratory

**Keywords:** Neurodata without borders, Neurophysiology, Data standardization, Scientific software

## Abstract

Modern neurophysiology generates increasingly complex, multimodal datasets that require standardized formats for effective sharing and reuse. The Neurodata Without Borders (NWB) format has emerged as a solution for data standardization, but data conversion remains a significant bottleneck due to format heterogeneity, metadata complexity, and required technical expertise. We present NeuroConv, an open-source Python library that automates the conversion of neurophysiology data from 47 distinct formats into NWB through a unified, modular architecture. Developed through collaboration with over 50 neurophysiology laboratories, NeuroConv addresses key challenges through three core components: format-specific DataInterfaces that abstract parsing complexity, multi-stream Converters that integrate heterogeneous data modalities, and optimized writing strategies for large-scale datasets including chunked operations and cloud-compatible storage. NeuroConv’s design enables researchers to convert complex, multi-modal experimental sessions with minimal code while preserving critical metadata and temporal alignment across recording systems. By removing technical barriers NeuroConv thus advances the transformation of neurophysiology toward FAIR (Findable, Accessible, Interoperable, and Reusable) data practices, facilitating reproducible research and accelerating scientific discovery.

## 1. Introduction

Modern neurophysiology produces complex, multimodal, large-scale datasets. Sharing this data has potential to accelerate the rate of scientific progress, but doing so effectively requires disciplined organization, annotation, and distribution to make it Findable, Accessible, Interoperable, and Reusable (FAIR) [1], [2]. However, the neurophysiology community faces significant challenges with sharing native data formats: the field’s diversity, spanning optical microscopy, electrophysiology, and behavioral tracking, involves multiple acquisition systems with formats often optimized for rapid data capture rather than shareability or portability. These formats vary widely in efficiency, metadata completeness and longevity of support, creating substantial barriers for data sharing [3], [4], [5].

The Neurodata Without Borders (NWB) format addresses these challenges and has become a leading solution, embedding rich contextual metadata directly alongside primary data to preserve interpretability over time [3], [5]. Crucially, NWB can accommodate the field’s diversity by uniting disparate modalities such as simultaneous behavioral and electrophysiological recordings typically stored in separate files and formats, thereby enabling rigorous re-analysis and bolstering reproducibility. Additionally, NWB architecture provides compression and chunking capabilities enabling efficient storage and data access even for large scale datasets.

Yet several factors still hinder broad NWB adoption. Converting neurophysiology data to any standardized format requires substantial technical expertise, from understanding diverse source format specifications to mastering the programming interfaces needed for data manipulation (pyNWB/MatNWB). Modern experimental setups amplify these challenges, as neurophysiology experiments now routinely combine multiple modalities (electrophysiology, imaging, optogenetics, behavior) each with unique format quirks, metadata particularities and synchronization needs. These technical barriers and resource requirements create a significant hurdle for NWB adoption.

Automated conversion pipelines address these challenges by eliminating the need for laboratories to repeatedly solve the same technical problems. Rather than requiring each research group to develop custom conversion scripts, automated tools provide standardized, tested pathways from diverse source formats to NWB. This approach not only prevents duplicated effort and inconsistent implementations across the field but also democratizes access to data standardization, ensuring that all laboratories can preserve and share their data regardless of available technical expertise. By lowering the barriers to NWB adoption, automated pipelines enable the neuroscience community to sustainably build a collective repository of standardized, reusable experimental data, ultimately accelerating scientific discovery through enhanced data sharing and reproducibility.

## 2. NeuroConv: Architecture and Design

To address the challenges described in Introduction (see Table 1), we developed NeuroConv, an open source library that automates the ingestion and conversion of neurophysiology data from diverse formats into NWB. This section describes in detail how NeuroConv’s architecture addresses each of these challenges through a modular, extensible design that maintains both flexibility and ease of use.

### 2.1. Handling Format Diversity

**Figure 1.**
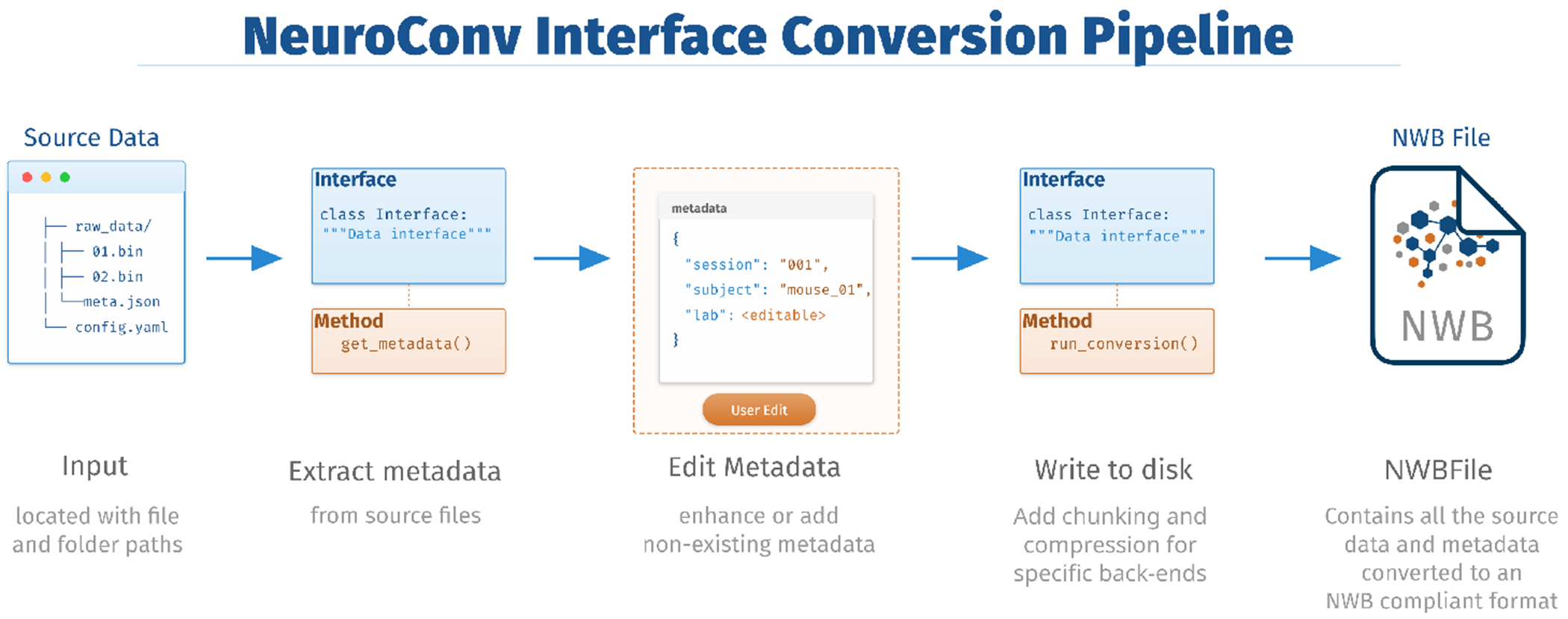
The process begins with source data (e.g., binary recordings, metadata, and configuration files). A data-specific DataInterface object is instantiated and used to extract metadata. The resulting metadata can be optionally edited by the user to fill in missing or corrected fields. The finalized metadata and source data are then processed to write a complete NWB file compliant with the standard

The challenge of format diversity in neurophysiology extends beyond their sheer number (47 supported at the moment). Many formats, such as Neuralynx, have multiple versions, while others, like TIFF, exhibit significant internal variability in how labs use them. NeuroConv addresses this complexity through a unified architecture centered on the DataInterface abstraction. DataInterface is an abstract class for reading data, and each supported format has a dedicated DataInterface that encapsulates the format-specific logic for data reading, metadata extraction, and NWB conversion while presenting a consistent API to users. The DataInterface serves as the fundamental building block of NeuroConv, providing a standardized pathway from diverse source formats to NWB output. This abstraction enables users to work with any supported format using identical code patterns, regardless of the underlying format complexity or internal details. The minimal conversion pipeline is illustrated in Figure 1:

Programmatically, this process can be summarized in a few lines of code, as shown below. The DataInterface handles all format-specific complexities internally, from parsing proprietary binary structures to extracting embedded metadata, while ensuring the output adheres to NWB best practices:

from datetime import datetime

from zoneinfo import ZoneInfo

from neuroconv import DataInterface

# Initialize the DataInterface for a specific format 

interface = DataInterface(file_path=“path/to/data/file”)

# Extract the metadata from the source format 

metadata = interface.get_metadata()

# Modify metadata as needed, add missing or correct existing fields

metadata[“NWBFile”][“experimenter”] = [“Baggins, Bilbo”]

metadata[“NWBFile”][“experiment_description”] = “Example neurophysiology experiment”

metadata[“NWBFile”][“institution”] = “University of Middle Earth”

# session_start_time is required for conversion

metadata[“NWBFile”][“session_start_time”] = datetime(2020, 1, 1, 12, 30, 0, tzinfo=ZoneInfo(“Middle-earth/Shire”))

# Add the data to an NWB File and write it to disk

interface.run_conversion(nwbfile_path=“path/to/nwbfile.NWB”, metadata=metadata)

This core pattern ensures consistency and simplicity across all supported formats:

1. Instantiate the appropriate DataInterface
2. Extract and modify metadata as needed
3. Execute the conversion

The DataInterface abstracts away format-specific complexities: from parsing proprietary binary structures to extracting embedded metadata.

Currently supporting 47 distinct input formats (table 2), each DataInterface is comprehensively documented and demonstrated in the Conversion Gallery, where users can find complete examples requiring only ~5 lines of code to perform full data conversion. Throughout the conversion process, NeuroConv enforces NWB Best Practices for metadata and data organization while optimizing data storage for both archival purposes and cloud computing requirements.

Each format example in our documentation includes basic code snippets that demonstrate how to use the DataInterface for that specific format, including metadata extraction, modification, and the conversion process. This modular approach allows users to easily adapt examples to their specific needs while providing a consistent interface across different data formats. For example, converting electrophysiology data acquired with Intan requires only these steps:

from datetime import datetime

from zoneinfo import ZoneInfo 

from pathlib import Path 

from neuroconv.datainterfaces import IntanRecordingInterface

file_path = f“{ECEPHY_DATA_PATH}/intan/intan_rhd_test_1.rhd” # This can also be .rhs

interface = IntanRecordingInterface(file_path=file_path, verbose=False)

# Extract what metadata we can from the source files

metadata = interface.get_metadata()

# session_start_time is required for conversion. If it cannot be inferred

# automatically from the source files you must supply one.

session_start_time = datetime(2020, 1, 1, 12, 30, 0, tzinfo=ZoneInfo(“US/Pacific”))

metadata[“NWBFile”].update(session_start_time=session_start_time)

nwbfile_path = f“{path_to_save_nwbfile}” # This should be something like: “./saved_file.nwb”

interface.run_conversion(nwbfile_path=nwbfile_path, metadata=metadata)

Supporting diverse data formats each with unique specifications, quirks, and version variations, represents a formidable technical challenge. To address this complexity, NeuroConv adopts a strategic approach: we leverage the collective expertise of the open-source neuroscience community by building upon established, well-tested libraries for format parsing. This philosophy recognizes that the neuroscience community has already invested considerable effort in developing robust parsers for specific data domains, and rather than duplicating this substantial work, we can build upon these proven foundations to create a more reliable and maintainable conversion ecosystem.

While we maintain specialized extractors for some formats (DeepLabCut, MedPC, FicTrac, etc), the majority of our DataInterfaces delegate the complex task of format parsing to domain-specific tools that have been extensively validated through widespread community use. DataInterfaces for electrophysiology utilize python-NEO [18] and SpikeInterface [19], which provide unified interfaces for raw and processed electrophysiology data, and ProbeInterface [20] for probe geometry metadata. For optical physiology, the NeuroConv team maintains ROIExtractors, which provides a unified API for optical physiology data across both raw imaging and processed segmentation outputs. ROIExtractors relies heavily on established libraries such as TiffFile [21]. Examples in behavioral data include the use of sleap-io for reading pose estimation data from SLEAP, SciPy [22] for audio, and OpenCV [23] for video.

Crucially, NeuroConv operates as an active contributor rather than a passive consumer of these dependencies. As our extensive usage of these libraries reveals bugs, performance bottlenecks, or functional limitations, we proactively engage with upstream developers by filing detailed, reproducible issue reports, submitting pull requests with tested fixes, contributing regression tests to prevent future breakages, and participating constructively in release planning discussions. This reciprocal development workflow ensures that improvements discovered during NWB conversion efforts propagate upstream, strengthening the broader neuroscience software ecosystem while continuously enhancing NeuroConv’s own reliability and robustness.

As NeuroConv’s format support has expanded to 47+ formats, dependency management has become increasingly complex. Each format often requires specialized libraries with potentially conflicting version requirements, creating dependency resolution challenges that can make installation difficult or impossible. For example, libraries for reading different formats may require different versions of Python or different versions of common dependencies such as numpy. Additionally, installing the dependencies of every DataInterface would create an unnecessary and inefficient environment with hundreds of packages, many of which users never need for their conversion.

To address these challenges, we rely on installation extras to manage installation complexity. Users can specify only the formats they need during installation:


pip install “neuroconv[spikeglx,phy,deeplabcut]”


This approach aggregates only the required dependencies for selected formats, avoiding dependency conflicts while ensuring that the installation remains lightweight and manageable for end users. Users working with a specific subset of formats can maintain clean, minimal environments without the overhead of unused dependencies.

### 2.2. Multi Stream Conversions

**Figure 2.**
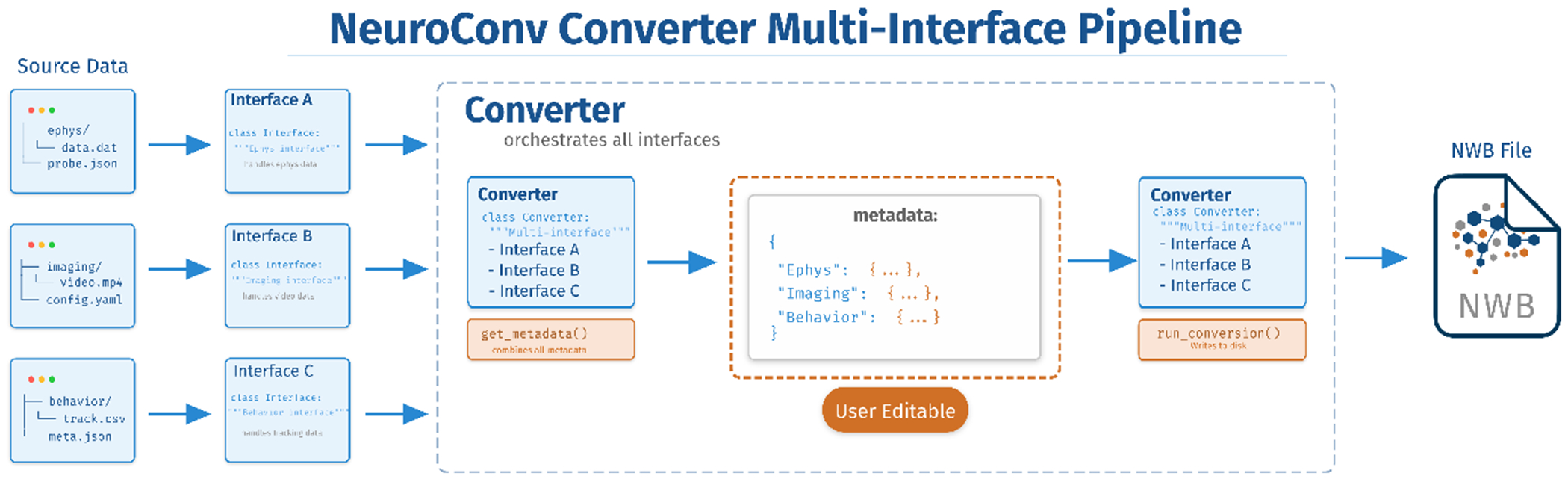
The Converter orchestrates multiple specialized interfaces (A, B, C), each handling different data types (electrophysiology, imaging, and behavior). Individual interfaces extract metadata from their respective data sources, which the Converter combines into a single, user-editable metadata structure. The Converter then coordinates all interfaces to produce a unified NWB file containing all data modalities. This design pattern enables flexible, modular integration of heterogeneous neuroscience data into a single standardized format.

Neurophysiology experiments typically involve multiple simultaneous data streams from different modalities, such as raw electrophysiology recordings, spike-sorted data, and behavioral video. Each stream may be stored in a different format, leading to complex conversion requirements. NeuroConv’s architecture supports multi-stream conversions through the aggregation of DataInterfaces within a Converter framework as illustrated Figure 2

The converter pattern enables combining multiple DataInterface instances into a single conversion workflow. This allows users to convert all relevant data streams from an experiment into a single NWB file, ensuring that all data is properly aligned and associated with the correct metadata. The Converter class handles the orchestration of multiple DataInterfaces, managing the order of operations and resolving any conflicts in metadata or data organization. Here’s an example of conversion for a multi-modal experimental session:

from datetime import datetime

from zoneinfo import ZoneInfo

from neuroconv import ConverterPipe

from neuroconv.datainterfaces import SpikeGLXRecordingInterface, PhySortingInterface, DeepLabCutInterface

# Initialize the DataInterfaces for each data stream

recording_interface = SpikeGLXRecordingInterface(file_path=“path/to/recording/file”) 

sorting_interface = PhySortingInterface(file_path=“path/to/sorting/file”) 

behavior_interface = DeepLabCutInterface(file_path=“path/to/behavior/file”)

# Create the ConverterPipe with the DataInterfaces

data_interfaces = [recording_interface, sorting_interface, behavior_interface] 

converter = ConverterPipe(data_interfaces=data_interfaces)

# Extract metadata from all interfaces 

metadata = converter.get_metadata()

# Modify metadata as needed, add missing or correct existing fields 

metadata[“NWBFile”][“experimenter”] = [“Baggins, Bilbo”]

metadata[“NWBFile”][“experiment_description”] = “Multi-modal neurophysiology experiment”

metadata[“NWBFile”][“institution”] = “University of Middle Earth”

# session_start_time is required for conversion

metadata[“NWBFile”][“session_start_time”] = datetime(2020, 1, 1, 12, 30, 0, tzinfo=ZoneInfo(“Middle-Earth/Shire”))

# Run the conversion to create an NWB file

converter.run_conversion(nwbfile_path=“path/to/nwbfile.nwb”, metadata=metadata)

Note that the same pattern used for single interfaces extends seamlessly to multi-stream conversions. The user initializes multiple DataInterfaces for each data stream, aggregates them into a ConverterPipe, extracts metadata, modifies it as needed, and then runs the conversion process to create an NWB file. This modular approach allows NeuroConv to handle complex experimental setups with multiple data streams while maintaining a consistent interface for users.

### 2.3. Multi-modal time synchronization

Precise temporal alignment across diverse recording modalities is essential for accurate multi-modal data analysis and reproducibility. NeuroConv streamlines this critical process by providing intuitive, unified methods for time synchronization, leveraging common temporal references like hardware clocks or synchronization pulses. NeuroConv provides convenience functions for extracting times from pulse signals, and for aligning time using a single starting pulse or for regular pulses sent between systems. These alignment strategies can correct for differences in starting time and for temporal drift between systems, and work for systems with very different sampling rates. This automation enforces best practices for temporal metadata in NWB and enhances downstream analysis integrity.

### 2.4. Handling High-Volume Data

Modern acquisition systems, such as multi-probe Neuropixel recordings or whole-brain optical imaging, generate massive volumes of data that continue to grow year over year [24], [25], [26]. These volumes of data pose a variety of challenges both for conversion and for long-term storage. Moreover, as cloud computing emerges as a solution [27], [28] for managing and storing large datasets, ensuring efficient accessibility for the scientific community becomes a critical consideration.

A critical feature is the ability to process datasets larger than available RAM. NeuroConv inherits from the work performed by the NWB core group with iterative writing to stream data in manageable chunks, with configurable buffer sizes based on available resources. We have extended this approach to support chunked reading that allows buffered writing of large datasets. This approach enables processing of arbitrarily large files and has been successfully tested on 100+ GB files using computers with only 8 GB of RAM.

For storage optimization, NeuroConv leverages chunking and compression in HDF5 and Zarr, the currently supported backends in NWB. Chunking allows large datasets to be read in manageable chunk sizes, and lossless compression algorithms allow us to reduce file size without altering the values of the data. There are many options for compression algorithms, and they present a trade-off between storage space and access speed [29]. NeuroConv exposes an easy-to-use API for configuring chunking and compression settings at the dataset level, allowing for quick experimentation while providing sensible defaults that work for most users.

Determining optimal chunk parameters involves complex tradeoffs [30]. Large chunks minimize the number of read operations but may require decompressing unnecessary data when accessing small subsets. Small chunks provide more precise access but increase overhead, particularly for cloud storage where each chunk requires a separate HTTP range request. Generally, appropriate chunking requires understanding the most common data access patterns. By understanding common analysis workflows and visualization patterns, it becomes feasible to implement evidence-based heuristics for chunk sizing across common data types, such as voltage recordings and imaging datasets.

### 2.5. Cloud Deployment

NeuroConv supports both local installation (Linux, Windows, or macOS) and cloud deployment through a maintained Docker image containing all dependencies. We’ve developed a YAML-based specification language for defining conversion pipelines, validated through JSON schema. This specification can fully describe multi-subject, multi-session conversions with custom metadata at each level, enabling automated conversion through containerized NeuroConv deployments.

### 2.6. Testing and Quality Assurance

Ensuring reliable conversion across diverse neurophysiology data formats requires a robust testing infrastructure. Our testing framework rests on two pillars: an automated continuous integration (CI) pipeline built using GitHub Actions and a comprehensive test data library.

Our continuous integration pipeline ensures code quality and maintains compatibility across operating systems through standard software engineering practices. The pipeline runs on every pull request and includes several key components: unit tests covering the core functionality of the library; integration tests ensuring the library works as expected with real data; cross-platform testing to verify compatibility across all supported operating systems; documentation builds to ensure up-to-date documentation; doctest functionality to verify that our conversion gallery works with the current version of the code, preventing documentation drift; code style checks ensuring consistency and adherence to best practices; and test coverage analysis to ensure the code is well-tested and maintainable. The code coverage of NeuroConv stands at 90%, which exceeds standard practices [31], [32]. Failed tests block pull request merging, maintaining code quality standards while facilitating rapid development.

Our test data infrastructure comprises carefully curated libraries spanning all supported formats, organized across three specialized domains: extracellular electrophysiology (developed in collaboration with NEO and SpikeInterface teams), optical physiology, and behavior. Each library contains representative files selected to encompass common usage patterns, format variations, edge cases, and diverse experimental configurations. This comprehensive coverage ensures that our testing captures real-world scenarios, from legacy format versions to modern acquisition systems with missing data streams or unconventional metadata structures. For continuous integration, we leverage G-Node’s git-annex technology for efficient large file management, maintaining lightweight repositories while providing full access to the test datasets. During continuous integration, GitHub Actions automatically downloads and caches these libraries, ensuring tests execute with current data while minimizing transfer overhead and maintaining rapid build times.

## 3. NWB Community and Software Ecosystem

### 3.1. NeuroConv and the NWB Conversion Landscape

NeuroConv occupies a strategic position within the broader NWB ecosystem, bridging the gap between low-level programming interfaces and high-level user tools. The conversion landscape offers different approaches suited to varying user needs and technical expertise levels.

At the foundational level, PyNWB and MatNWB provide direct programmatic access to the NWB specification, offering maximum flexibility but requiring deep understanding of the standard. NWB GUIDE offers a graphical interface that democratizes access to NWB conversion through guided workflows, making it accessible to users without programming experience. NeuroConv complements both approaches by providing programmatic automation while maintaining the flexibility needed for complex, multi-modal experimental setups. This ecosystem approach ensures that researchers can choose the conversion method that best matches their technical expertise and experimental complexity, while all approaches converge on the same standardized output format.

### 3.2. Integration with Data Archives and Visualization

The Distributed Archives for Neurophysiology Data Integration (DANDI) Archive complements NWB by providing free hosting for terabyte-scale datasets, letting researchers satisfy NIH data-sharing mandates while archiving data in a versioned, API-driven repository connected to a rich ecosystem of visualization and analysis tools. NeuroConv integrates tightly with DANDI through automated upload functionality that validates required metadata, enforces DANDI naming conventions, and automatically uploads NWB files to the archive. Together, NWB, NeuroConv, and DANDI showcase a comprehensive pipeline that spans the entire data lifecycle from acquisition to publication and reuse, supporting emerging software domains from electrophysiological spike sorting to calcium imaging segmentation and behavioral pose estimation.

## 4. Current Limitations

While NeuroConv has significantly improved data standardization processes, some major challenges remain:

### Format Coverage:

Despite supporting 47 formats, the neurophysiology ecosystem continues to evolve with new acquisition systems, format versions, and processing software. Each new system often introduces proprietary formats with unique metadata structures and data organization schemes. While NeuroConv’s modular architecture allows users to develop custom DataInterfaces for unsupported formats, this process requires substantial technical expertise in both the source format’s internal structure and NeuroConv’s interface architecture. Additionally, maintaining custom interfaces as both the source format and NeuroConv evolve presents ongoing maintenance challenges for individual laboratories.

### Custom Laboratory Formats:

Many laboratories develop custom data storage solutions tailored to their specific experimental workflows, often utilizing MATLAB files, custom CSV schemas, or bespoke binary formats. These custom formats frequently exhibit significant variability not only between laboratories but even within the same laboratory over time as experimental protocols evolve. The heterogeneous nature of these formats, ranging from simple tabular data to complex nested structures with laboratory-specific metadata conventions, makes automated conversion particularly challenging. While NeuroConv can handle some standardized custom formats, the diversity of these approaches often requires manual intervention or custom code development. For these cases, we provide documentation for building custom DataInterfaces that build upon our established abstractions and integrate directly with other DataInterfaces in multi-modal Converter workflows. Since users understand their own data formats best, they are well-positioned to develop these interfaces. This approach is particularly valuable for behavioral data where standardization remains elusive [33].

### Metadata Completeness:

While NeuroConv automatically extracts available metadata from source formats, many acquisition systems store incomplete or inconsistent metadata. Critical information such as experimental conditions, subject details, or calibration parameters may be missing, incorrectly formatted, or stored in non-standard locations. This limitation requires manual metadata curation and validation, which can be time-consuming for large datasets. Additionally, some formats store metadata in proprietary or undocumented structures that may not be fully accessible through existing parsing libraries.

### Complex Experimental Paradigms:

Highly specialized experimental setups such as those involving custom-built hardware, novel stimulation protocols, or unique behavioral paradigms, may not map directly onto NWB’s data organization schemes. While NWB provides extension mechanisms for custom data types, leveraging these extensions through NeuroConv requires additional development effort and deep understanding of the NWB specification. These edge cases often require manual intervention or custom conversion pipelines that may not be easily generalizable to other laboratories.

### Programming Prerequisites:

While NeuroConv substantially reduces the coding burden, it still requires basic programming knowledge, including object-oriented concepts. While we aim to make the library as user-friendly as possible, users must still be comfortable with python programming to effectively utilize NeuroConv. This includes understanding how to install the library, manage dependencies, and write scripts that leverage the DataInterface classes for conversion.

## 5. Future Directions

### Enhanced User Experience:

We are implementing comprehensive improvements to examples, tutorials, and documentation. We aim to slowly transition to the Diataxis [34] framework which will provide clearer learning pathways for users with different backgrounds and goals.

### NWB Standard Evolution:

We actively participate in NWB schema development and maintain close alignment with emerging standards through engagement with NWB Extensions Proposals. By monitoring and contributing to current developments, including enhanced schemas for experimental events (NWBEP001), extracellular electrophysiology (NWBEP002), and optical physiology (NWBEP003, NWBEP004), we ensure that NeuroConv incorporates the latest standard improvements as they become available for the benefit of our users.

### AI-Assisted Conversion:

We are exploring large language model integration to advance our core mission of automating neurophysiology data conversion. More concretely, we are developing an LLM-powered conversion agent that uses retrieval-augmented generation over neuroconv documentation and curated examples to synthesize NWB-compliant conversion code and validate the results with nwb-inspector. Grounded in verifiable sources, the agent streamlines converter creation while minimizing hallucinations and ensuring reproducibility.

### Cloud-Optimized Storage:

We are implementing improved chunking patterns and compression strategies for large datasets to enhance cloud access performance. This includes systematic experimentation through the NWB Benchmarks Project to determine optimal chunk sizes and compression algorithms [29]. Our goal is to implement evidence-based heuristics that ensure efficient and performant data storage, as well as having a friendly and clear API that allows customization and flexibility for users to adapt to their specific needs. In the future, we would also like to explore the inclusions of cost optimization considerations for cloud storage [35].

## 6. Conclusion

We believe that NeuroConv represents a critical step toward realizing the vision of FAIR neurophysiology data. By automating the conversion of diverse data formats into a common standard, we enable researchers to focus on scientific discovery rather than data wrangling. The success of this approach depends not only on technical implementation but on fostering a community that values standardization, reproducibility, and open science.

Our work demonstrates that effective scientific software development requires balancing automation with flexibility, standardization with customization, and ease of use with powerful capabilities. The challenges we have addressed such as format diversity, metadata complexity, and scale are not unique to neurophysiology but represent broader issues in scientific computing that require community-driven solutions.

The broader implications extend beyond neurophysiology to any scientific domain grappling with data heterogeneity and the need for standardization. We believe that our approach of abstracting format complexity through unified interfaces, while maintaining extensibility through modular architecture, provides a template for similar challenges in other fields.

As the neurophysiology community continues to generate increasingly complex and large datasets, tools like NeuroConv become essential infrastructure for scientific progress. By lowering barriers to data standardization and sharing, we contribute to a future where scientific data is truly FAIR, accelerating discovery and enhancing reproducibility across the field.

## Figures and Tables

**Figure 3.**
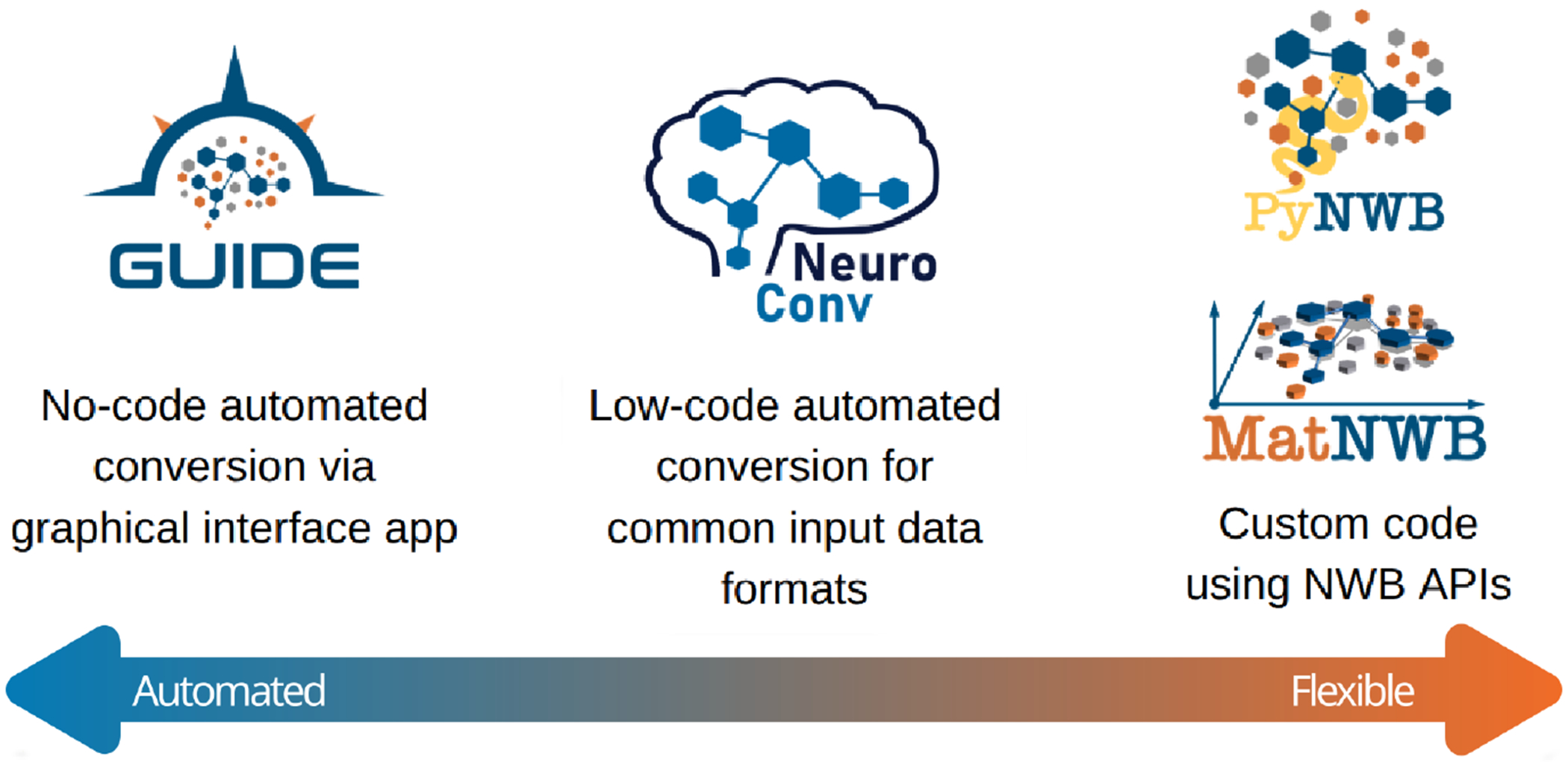
This illustrates where NeuroConv stands in regard to other conversion tools. For low-level, high-precision control, users can employ the NWB apis directly (PyNWB in Python, MatNWB in MATLAB). For a guided GUI-based experience, the NWB GUIDE offers the best option but may be too rigid for complex workflows. NeuroConv stands as a middle ground, automating the conversion of a large number of formats while still allowing for customization and flexibility.

**Table 1.** Key challenges in building automated conversion pipelines to the NWB format.

Challenge	Description
Format diversity	Source data formats span both proprietary and open standards, with many formats existing in multiple versions and internal variations
Metadata complexity	Metadata requirements vary substantially across experimental paradigms, with critical information often stored inconsistently or incompletely
Scale challenges	Dataset sizes frequently reach hundreds of gigabytes to terabytes, requiring specialized handling for memory-efficient processing
Multi-modal integration	Experiments often combine multiple recording systems (electrophysiology, imaging, behavior) each storing data in different formats. This also introduces the problem of data alignment and synchronization across modalities.

**Table 2.** Comprehensive list of data formats supported by NeuroConv, organized by experimental modality and data type.

Modality	Sub-modality	Format
**Extracellular Electrophysiology**	*Recording*	AlphaOmega Axona Biocam Blackrock European Data Format (EDF) Intan MaxOne MCSRaw MEArec Neuralynx NeuroScope OpenEphys Plexon Plexon2 Spike2 Spikegadgets SpikeGLX Tucker-Davis Technologies (TDT) White Matter
	*Sorting*	Blackrock Cell Explorer ([6]) KiloSort ([7]) Neuralynx NeuroScope Phy Plexon
Intracellular Electrophysiology	—	ABF
**Optical Physiology**	*Imaging*	Bruker HDF5 Micro-Manager Miniscope ([8]) Scanbox ScanImage Thor Tiff
	*Segmentation*	CaImAn ([9]) CNMFE ([10]) EXTRACT ([11]) Minian ([12]) Suite2P ([13])
	*Fiber Photometry*	TDT Fiber Photometry
**Behavior**	*Motion Tracking*	DeepLabCut ([14]) FicTrac ([15]) LightningPose ([16]) Neuralynx NVT SLEAP ([17])
	*Audio/Video*	Videos
	*Operant Conditioning*	MedPC
**General Data**	*Image*	Image (png, jpeg, tiff, etc)
	*Text/Tabular*	CSV
		Excel
